# Open access and digital morphology data in evolutionary biology: expanding frontiers of knowledge

**DOI:** 10.1186/s12862-026-02522-y

**Published:** 2026-04-28

**Authors:** Naomi De Leo, Margot Michaud, Luigi Maiorano, Carlo Meloro, Narimane Chatar, Davide Tamagnini

**Affiliations:** 1 Department of Biology and Biotechnologies “Charles Darwin”, Sapienza University of Rome, Rome, Italy; 2 IDEALISS ULR 7519, Institut Polytechnique UniLaSalle, Université d’Artois, Mont-Saint-Aignan, France; 3 Département Formation et Recherche Sciences et Technologie, Université de Guyane, Cayenne, French Guiana; 4 Research Centre in Evolutionary Anthropology and Palaeoecology, School of Biological and Environmental Sciences, Liverpool John Moores University, Liverpool, UK; 5 Department of Integrative Biology, UC Berkeley, Berkeley, CA USA; 6 Departamento de Ecología y Geología, Universidad de Málaga, Málaga, 29071 Spain; 7 Evolution and Diversity Dynamics Lab, Université de Liège, Liège, Belgium

**Keywords:** Open access, Evolutionary biology, Digital morphology, FAIR principles, 3D morphometrics

## Abstract

The recent integration of 3D imaging and digital methodologies has revolutionized evolutionary biology, offering unprecedented opportunities for analysing and sharing morphological data. However, the transition toward open access remains incomplete due to persistent technical, legal, and institutional barriers. Issues such as lack of standardization, massive file sizes, and unclear intellectual property rights continue to hinder data verification and reproducibility. These challenges have acquired new urgency with the rapid rise of machine learning and AI-based tools for automated segmentation, landmarking, and shape analysis, which require large, standardized, and openly accessible training datasets — making inaccessible 3D data not merely an inconvenience, but a source of systematic bias in the algorithms shaping the field’s future. This review synthesizes technical, legal, and behavioural perspectives on open data in digital morphology, building on prior work to address the specific challenges of the current AI era. By advocating for the adoption of FAIR principles, the use of persistent digital identifiers, and the implementation of digital watermarking, we offer recommendations for establishing minimum standards in data publication. Ultimately, a shift toward responsible data stewardship is essential to ensuring that digital morphological resources remain accessible, reproducible, and scientifically valuable for both human and computational users.

## Background

**Fig. 1:**
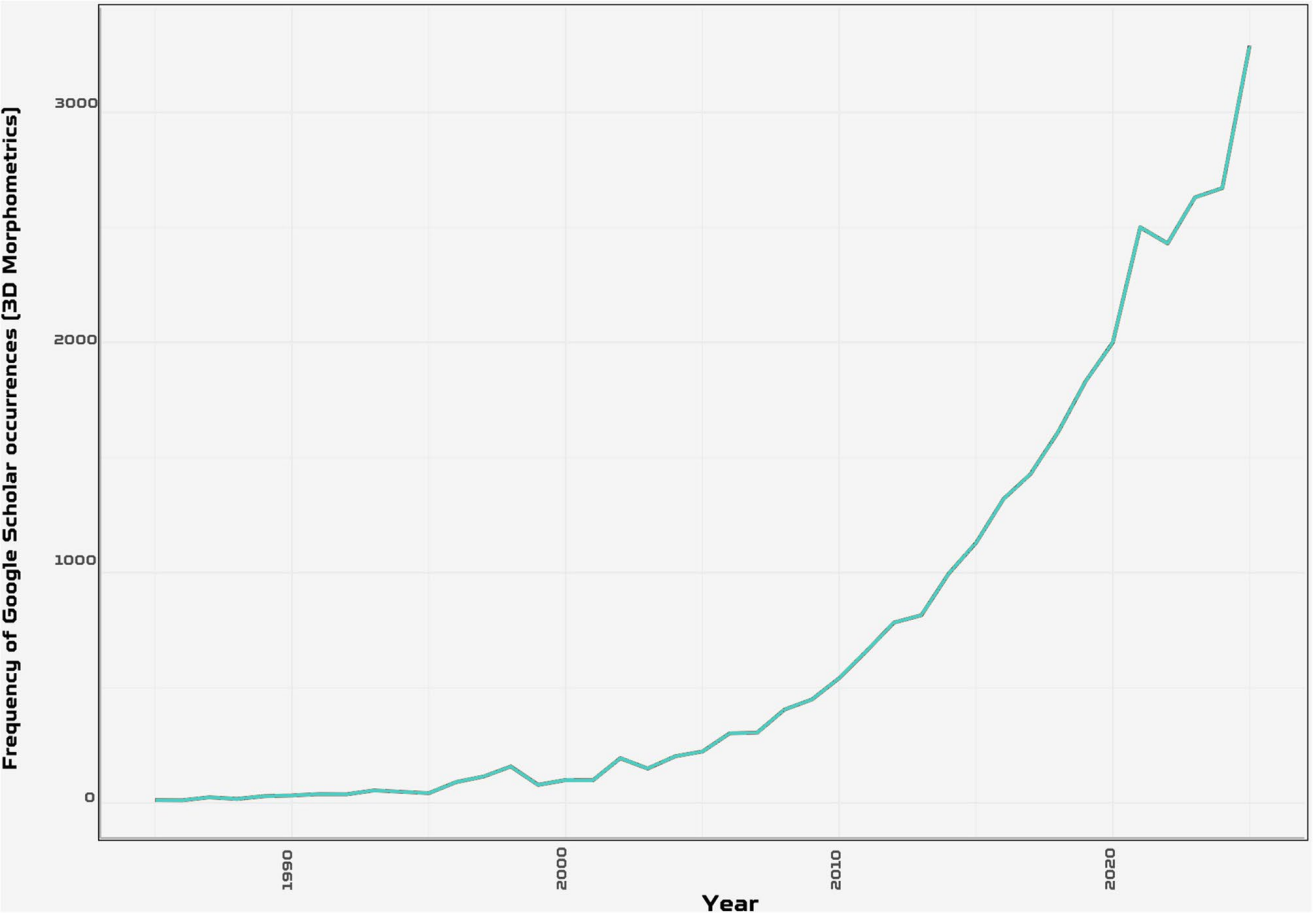
Frequency of Google scholar occurrences “3D morphometrics” (1990–2025) on 28 March 2026, showing exponential growth

**Fig. 2:**
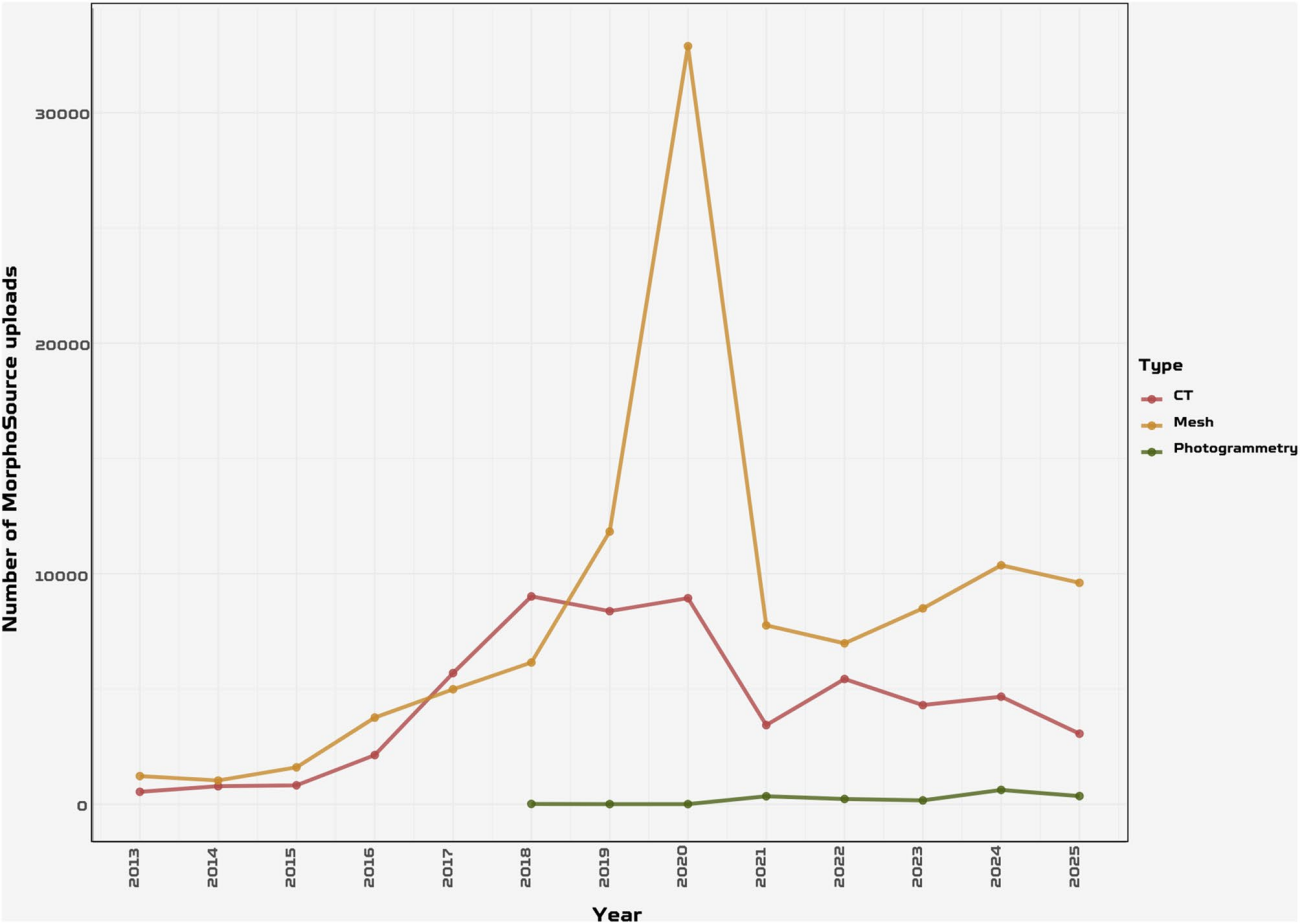
Number of uploads to MorphoSource by data type (CT scans, mesh files, photogrammetry) from 2013 to 2025. A spike in mesh file uploads occurred in 2020, coinciding with the COVID-19 pandemic and likely reflecting increased researcher availability for data processing and sharing. Mesh uploads remained elevated post-pandemic. Data downloaded from MorphoSource in March 2026

In recent years, the evolutionary biology community has witnessed a revolutionary transformation towards digital methodologies, 3D scanning and quantitative approaches, redefining the research landscape across various disciplines [1–5]. The development of new methods for rapid or automated processing of imaging data [6–11], greater accessibility of 3D scan [12–15], and new methods for quantifying anatomical data [16–19] have made it more popular than ever to apply complex quantitative approaches for the study of phenotypic diversity, even inflating the volume of datasets (Fig. 1). This exponential growth was further accelerated by the COVID-19 pandemic, which simultaneously increased researchers’ capacity to upload existing datasets and created an urgent demand for remote access to 3D specimens as physical collections became temporarily inaccessible worldwide [14]. The sharp increase in MorphoSource uploads observed in 2020 (Fig. 2) reflects how external pressures can rapidly shift community behaviour toward open data practices. This spike during the COVID-19 pandemic also illustrates a persistent challenge with 3D data: that their handling and processing can be time-consuming (e.g., standardising 3D models and making them available online); dynamic with important implications for future policy interventions. Fortunately, the recent improvements in 3D databases such as the batch upload tool in MorphoSource make the deposition of 3D models much easier. In palaeontology and evolutionary biology, this digital transition has introduced significant innovations [20–23], notably through the rapid development of digital morphometrics, which has expanded the analytical and collaborative potential of morphological research by enabling more rigorous data sharing, multiscale visualization, and reproducible quantitative analyses [14, 23, 24].

In particular, geometric morphometrics (GMM) includes both two-dimensional (2D) and three-dimensional (3D) approaches that employ digital imaging techniques to extract morphological data from biological specimens [24–27]. While 2D geometric morphometric analyses can be performed on photographs, 3D GMM makes use of digital reconstructions generated through methods such as CT scanning, surface scanning, or photogrammetry. These imaging techniques allow researchers to build high-resolution 3D models of specimens, which serve as the basis for quantitative shape and size analysis. In the last decade the adoption of these digital tools has expanded the analytical potential of morphometric research by enabling the extraction of more detailed and accurate morphological information, particularly in the study of complex anatomical structures that are difficult to capture with traditional 2D approaches or linear measurements [27–30]. Importantly, 3D datasets preserve the true proportions and spatial relationships of anatomical traits, avoiding the significant shape distortions, such as parallax errors and perspective effects, that occur when complex 3D structures are projected onto a 2D plane [31]. This is particularly critical for vertebrate skulls, where 2D photography can fail to capture the depth of the zygomatic arches or the exact curvature of the braincase, leading to an underestimation of biological variation [32].

Furthermore, open access digital datasets can enhance methodological development by improving data accessibility, enabling more detailed analyses, and supporting transparency and reproducibility [33, 34]. This approach promotes broader collaboration, reproducibility of analyses, and innovation in morphometric applications across different research fields [25, 35–37].

While digital datasets in anthropology, zoology, and palaeontology represents scientific areas scenario undergoing profound transformation [14, 25, 38, 39], examples from other disciplines underscore the potential of open data sharing on consolidated and well-established repositories. In genetics the open dissemination of genomic sequences has revolutionized research, enabling breakthroughs in evolutionary biology, medicine, and biotechnology. Related initiatives such as GenBank [40] and the Human Genome Project [41] illustrate how centralized, standardized, and openly accessible datasets can catalyse scientific advancements, fostering interdisciplinary collaboration and accelerating discovery rates [1, 42, 43]. Similarly, in the field of palaeontology, the Paleobiology Database (PBDB) stands as a prime example of a community-driven effort to centralize scientific data. PBDB reflects decades of global collaboration that has led to a clear and demonstrable advantage in data accessibility. As recently highlighted by Dowding et al. (2026) [44], the PBDB represents a rare ‘success story’ of digital infrastructure longevity in a landscape where 95% of databases fail within 15 years due to funding instability. 

Despite these advancements, open access data sharing in the realm of digital morphology in comparative and evolutionary biology presents a complex array of challenges and opportunities warranting thorough exploration. Issues surrounding data privacy, intellectual property rights, and the long-term sustainability of digital repositories are becoming increasingly relevant, alongside technical challenges related to data standardization, quality, and accessibility [25, 38].

In this context, the transition toward open access can be viewed as a “challenging triangle” of interconnected barriers: technical, legal, and psychological ones. The technical aspect involves the management of massive datasets and the lack of universal standards for 3D metadata [25], while the legal and institutional aspect are complicated by unclear intellectual property rights and the risk of commercial exploitation of digital derivatives [45]. Finally, these barriers are reinforced by a psychological aspect, a cultural resistance rooted in the fear of data misuse or loss of academic priority [38].

**Fig. 3:**
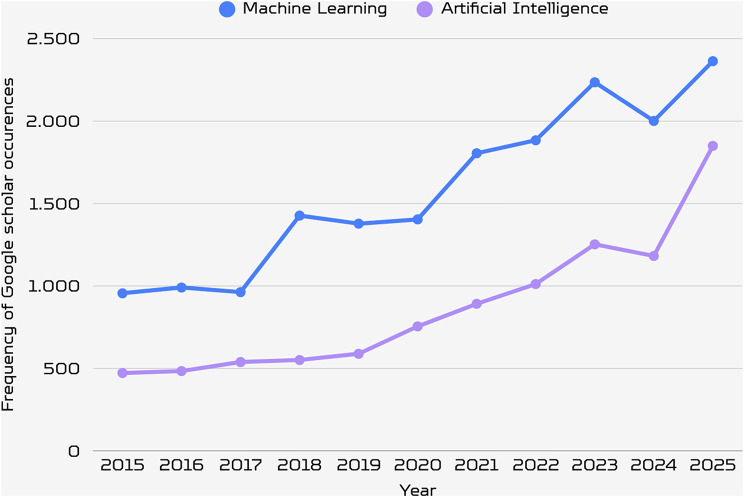
Frequency of Google scholar occurrences for “Machine Learning” (a specific methodology) and “Artificial Intelligence” (a group of methodologies) combined with “geometric morphometrics” or “paleontology” from 2015 to 2025 on 28 March 2026. Both terms show substantial growth, with machine learning consistently more prevalent, probably because is a more specific terminology. A marked acceleration occurred from 2018 onward, reflecting increasing integration of computational approaches in morphological research.

Given these long-standing challenges, why has it become more urgent than ever to implement the open data standards proposed by prior reviews? The answer lies in the rapid rise of machine learning and AI-based tools in morphological analysis. The exponential growth of these applications (Fig. 3) has introduced a qualitatively new dimension to the open data problem: inaccessible or poorly standardized 3D datasets no longer merely slow individual research; they risk systematically biasing the automated pipelines that are increasingly defining the field. Also, the legal landscape around 3D derivatives is rapidly evolving worldwide, raising new copyright questions that earlier reviews could not anticipate. This review addresses these gaps by integrating technical, legal, and behavioural perspectives into a unified framework designed for this new wave of data-driven morphological research.

This review aims to investigate the current state of open access data and the role of digital morphology in zoology, ecology, macroevolution, and scientific museology. It will first examine the main imaging techniques and types of digital output currently used, followed by an overview of their applications in research contexts, including evolutionary studies, ecological analysis, and educational or curatorial use. We will focus on the most pressing problems related to data sharing in digital morphological research, such as repository sustainability, lack of standardization, and copyright restrictions. Finally, we propose a range of potential solutions and recommendations, including the implementation of FAIR principles, digital watermarks, and improved citation practices.

Although 2D geometric morphometrics has long been used in evolutionary and ecological studies, this review focuses primarily on 3D data and techniques. This choice reflects both the transformative impact of 3D digital assets in modern morphology and the unique, escalating challenges they present for open sharing; particularly regarding file size and complexity [25, 46], digital rights [45, 47], and repository sustainability [48]. Unlike 2D datasets, 3D models are increasingly treated by institutions as high-value ‘digital twins’ with complex legal and economic implications [49]. While 2D datasets provide valuable information and insights, they capture a limited number of aspects of morphology compared to 3D models, which offer enhanced anatomical details and immersive interactivity, making them especially useful for replication, reanalysis, and educational outreach [25, 28].

Furthermore, the urgency of addressing 3D data management is driven by the rise of additive manufacturing (3D printing) and the integration of 3D datasets into Machine Learning (ML) and Artificial Intelligence (AI) frameworks [50]. These technologies, ranging from automated taxon identification to generative modelling, require high-resolution volumetric data, making the 3D format a critical ‘battleground’ for current Intellectual Property Rights (IPR) debates. Consequently, while the open sharing of 2D data is traditionally constrained by museum policies regarding image dissemination [45, 51], 3D morphological assets present a distinct and more complex set of challenges that this review addresses in detail.

As this digital transition continues to reshape scientific practice, critically addressing the barriers and opportunities of open data in digital morphology is no longer optional, it is essential to unlock the full potential of morphological knowledge and ensuring its legacy for future research.

## Main techniques

In zoology, and phenotypic analysis on vertebrates in particular, the adoption of digital morphology-based techniques, especially three-dimensional (3D) imaging, has revolutionized the way researchers analyse and interpret the morphological features of biological specimens [25, 52, 53]. These digital techniques enable the non-invasive exploration of morphological traits by preserving the integrity of biological specimens while offering unprecedented access to high-resolution, morphologically complex features that were previously inaccessible [25, 54]. Crucially, when these outputs are made available through open-access platforms (e.g. MorphoSource, Digimorph, MorphoMuseuM, etc.), they allow other researchers to reanalyse specimens virtually, overcoming the logistical and curatorial constraints often associated with physical collections.

One of the main techniques in this field is photogrammetry, which stands out due to its affordability and high-quality outputs [25, 53, 55]. By stitching together multiple overlapping photographs taken from various angles, photogrammetry constructs detailed 3D models of preserved specimens. This technique requires minimal equipment, often just a standard camera and a computer [56]. Recent improvements in modern smartphone cameras now allow users to produce low resolution photogrammetry models [57, 58], making it widely applicable not only in palaeontology [56] but also in types of natural history collections (e.g., [30]). The resulting models provide a digital archive of specimens that can be manipulated and examined in ways physical specimens cannot, such as virtual dissections or volume measurements.

Surface scanning, including both handheld and stationary setups, offers another robust method for capturing the surface details of specimens [25, 59]. These devices project a laser flash onto the surface of an object and measure the time delay or displacement of the reflected light, which is then used to generate precise 3D models. This technique is highly valued for its accuracy and the ability to capture fine-grained surface, which are crucial for detailed morphological studies [53, 60]. However, surface scanners are more complex to learn in detail, tend to produce large datasets and may require proprietary software (e.g., Artec Studio), which can complicate sharing in open-access formats unless standardized export protocols are used.

CT scanning, or computed tomography, uses x-rays to create cross-sectional images of objects from which digital 3D models are reconstructed [25, 61]. Unlike surface scanning techniques, CT scanning provides insights into the internal structure of specimens, non-destructively revealing details about skeletal architecture, pathology, and/or developmental stages. This method is critical for studying specimens that are either too fragile to handle (e.g., fossils) or whose internal morphology provides significant insights into phylogenetic placement, functional adaptations, or ontogenetic changes [54].

The open access movement significantly amplifies the impact of the previously mentioned technologies by facilitating the global sharing of digital morphological data [25, 53, 62]. The difficulty of sharing is usually proportional to the storage size of the raw data, which depends primarily on acquisition parameters such as image resolution, number of reconstructed slices, and bit depth. These in turn reflect the level of anatomical detail required by the research question, rather than the intrinsic complexity of the specimen. While a simplified 3D mesh may be easily hosted on public repositories, the raw data, essential for full scientific transparency and re-analysis, often poses a significant burden on storage infrastructure and bandwidth [63], creating a “data bottleneck” that varies significantly across techniques (see Table 1). By raw data we refer to the primary acquisition outputs specific to each technique: x-ray projections and reconstructed image volumes for CT scanning, photographs and point clouds for photogrammetry, and point clouds for laser scanning; all of which are substantially larger than derived mesh files but necessary for full reproducibility.

By overcoming these barriers, researchers from around the world can access, analyse, and compare 3D models, contributing to a more comprehensive understanding of evolutionary biology and enhancing the reproducibility of scientific research. Moreover, open access to digital morphological data supports interdisciplinary studies, allowing experts in fields such as biomechanics, paleoecology, and evolutionary biology to collaborate effectively [52]. This entanglement is further strengthened by centralized databases and open platforms where researchers can upload and exchange their digital collections, thereby nurturing a rich, communal resource that accelerates scientific discovery and education in palaeontology and beyond [25, 62].

Nonetheless, the effectiveness of these platforms depends critically on sustained institutional support, standardization of metadata, and proper citation practices, factors that are often uneven across current repositories. Recognizing these constraints is essential for ensuring that digital morphology remains both scientifically valuable and broadly accessible [38].

**Table:** 

Box 1.1 - Surface scanning techniques and typical 3D file formats	
In the last two decades multiple types of surface scanners have become widely used for data collection in comparative and evolutionary biology. Laser scanners, for instance, measure the time delay or displacement of reflected laser beams to create a 3D model, recording depth information at various points on the surface through a sensor (usually a Charge-Coupled Device camera), following calibration via triangulation [64]. By repeating the scanning process from multiple angles, the scanner uses overlapping surfaces to align and stitch individual scans, producing a complete 3D object. This data is then converted into 3D coordinates (x, y, and z) using the scanner’s coordinate system [65]. Another common surface scanning method is structured-light scanning, which projects patterned light onto the object. If the surface is planar and lacks 3D variation, the pattern captured by the camera closely resembles the projected one. However, when the surface is non-planar, its geometry distorts the projected pattern as seen by the camera [66]. Both laser and structured-light scanners have their advantages and limitations: laser scanners generally offer higher resolution, but recent work has shown that structured white-light scanners with a broad field of view can also produce accurate models through flat glass [67]. Unlike photogrammetry, which requires minimal investment, surface scanning entails significant costs: not only for the scanner itself but also for ongoing software license fees needed to process raw scan data. The resulting 3D meshes are saved in different file formats, each with specific characteristics. The most common formats include PLY (Polygon File Format or Stanford Triangle Format), STL (Polygon File Format or Stanford Triangle Format) or OBJ. These formats offer different ways of encoding essential geometric data such as vertices, faces, and sometimes texture coordinates. The PLY format was developed at Stanford University and is widely used in research and academic contexts. It can store rich information, including colour and transparency values, and supports both ASCII and binary encoding. The STL format is one of the oldest and most widely used formats, particularly in 3D printing. Despite its technical limitations, such as the inability to store scale, colour, or material information, the STL format remains the de facto universal standard in digital morphology. Its simplicity ensures that every software suite used for 3D modelling, analysis, or printing can read and process these files, making it a crucial bridge for data interoperability. The OBJ format, developed by Wavefront Technologies, is commonly used in computer graphics and animation. It supports polygonal geometry and can include texture maps, UV coordinates, and material properties, making it ideal for applications that require detailed visual rendering. Some software programs require specific formats (e.g., STL, as mentioned above, is very common in 3D printing software). OBJ files can embed texture references directly within the file (textured OBJ), making them convenient for visualization purposes. In contrast, STL and PLY formats typically require an additional file (e.g., JPG or PNG) to store texture information, and PLY is not well-suited for handling textures. On the other hand, PLY files are often more efficient in terms of storage, as they can represent the same 3D models using less disk space, especially when saved in binary format.	

**Table 1:** Comparative analysis of data volume and storage requirements across digital morphological acquisition techniques. The table illustrates the “data iceberg” effect in 3D digitization, showing the discrepancy between raw project files (Input) and finalized 3D meshes (Output)

Methodology	Raw/Project Data (Input)	Final 3D Model (Output)	Storage & Sharing Complexity
**2D Morphometrics**	**~** **10**–**50** **MB** (High-res Photos)	**< 1 MB** (Landmark Coordinates)	**Low.** Only coordinates are usually shared.
**Photogrammetry**	**~ 800 MB – 1 GB** (Set of 100 + RAW/JPG photos)	**from 2 MB to ~ 100 MB** (Textured OBJ/PLY)	**Medium.** Photos are usually not shared for dimension.
**Surface Scanning** (e.g., Artec Spider)	**~ 10 GB – 20 GB** (Raw point clouds & scans)	**~ 100 MB – 2 GB** (Simplified STL/PLY mesh)	**High.** Projects are massive to download/upload.
**CT / Micro-CT Scanning**	**~ 400 MB – 50 GB+** (DICOM stacks/TIFFs)	**~ 30 MB – 500 MB** (Segmented STL/PLY mesh)	**Very High.** E.g. DICOM/TIFF data is essential for re-segmentation but requires massive bandwidth.

## Applications in research, education, and institutions

Building on the increasing use of digital imaging techniques and open-access platforms, morphological datasets are now transforming multiple domains, including both research fields and institutional settings such as museums and education. In research, the growing availability of high-resolution 3D morphological data is revolutionizing disciplines like ecology, morphometrics, palaeontology, and macroevolution by enabling large-scale, reproducible analyses of organismal form and function. Traditionally, morphological studies have relied on physical specimens housed in museum collections, limiting access and data integration across disciplines. The shift towards digitalization has removed many of these barriers, allowing the creation of high-resolution 3D models that can be analysed, shared, and reused across diverse research contexts [68, 69]. This was proven particularly useful during the recent Covid pandemic, where researchers from all around the world could access 3D models of specimens housed on public repositories [14]. This section discusses these diverse impacts, highlighting advances in data acquisition, analytical applications, and the ethical considerations surrounding open digital morphology.

In trait-based ecology, open-access digital datasets enhance the ability to quantify functional traits and model species-environment interactions [70, 71]. The availability of 3D models has facilitated more precise simulations of key eco-evolutionary dynamics—such as predation, locomotion, and thermoregulation—while also reducing the ethical concerns associated with live animal experimentation [68, 70]. The incorporation of colour mapping (i.e., textures) into digital datasets represents a promising frontier, as coloration provides essential data for behavioural and adaptive studies, such as aposematism and sexual selection [68]. Coloration studies are predominantly conducted using 2D digital imaging, which allows for precise colour calibration and standardized backgrounds necessary for consistent analysis. For instance, MacLean et al. (2018) [72] utilized 2D digital images of museum specimens of the butterfly *Colias meadii* to detect historical changes in wing melanism over 60 years, demonstrating how digital phenotypic data can reveal climate-linked trends in functional traits. While current 3D datasets focus primarily on shape, the integration of high-fidelity colour textures into 3D models remains less common due to challenges in standardized colour calibration across complex geometries. This is most relevant for surface-based techniques such as photogrammetry and structured light scanning, which directly capture colour information. For volumetric techniques such as CT, MRI, or synchrotron imaging, colour data can only be added through co-registration with surface scans or photogrammetric models of the same specimen. Advancing this synergy could unlock new research possibilities, allowing for a more complete digital representation that combines volumetric precision with functional colour data — particularly relevant for studies of coloration-dependent traits such as camouflage, sexual selection, or species identification.

Beyond the digital preservation of specimens, these high-resolution 3D assets are pivotal for multiple computational pipelines that go beyond traditional shape analysis. For instance, detailed surface meshes and volumetric data are essential for Finite Element Analysis (FEA), allowing researchers to simulate and investigate the biomechanical performance of complex structures, such as cranial joints or dental apparatuses. Similarly, 3D imaging enables the digital reconstruction and ‘virtual peeling’ of internal cavities (e.g., endocasts), providing access to neuroanatomical data that would otherwise remain hidden. Furthermore, the burgeoning field of machine learning in morphology is opening new avenues for automated taxon identification and landmark placement [50]. By leveraging large-scale 3D datasets, these automated approaches significantly increase the reproducibility and throughput of evolutionary studies, making GMM the ideal framework for examining the current challenges of Open Access and data standardization.

In macroevolution, the use of digital morphological data supports comparative analyses across broad temporal and taxonomic scales with the implementation of phylogenetic comparative methods (PCMs). These statistical approaches account for shared evolutionary history among species when analysing trait data, allowing researchers to make more accurate inferences about evolutionary patterns and processes [73]. The integration of PCMs and morphological datasets has fostered a synergistic relationship, not only improving the resolution of ancestral state reconstructions but also providing a more robust framework for analysing evolutionary paths [24, 74]. Crucially, open-access 3D repositories significantly expand taxonomic coverage—especially in paleontological research, where direct access to fossil specimens across museum collections around the world is often logistically and financially impossible to be realized [38]. This expanded coverage enhances the robustness of macroevolutionary models, allowing for a more accurate reconstruction of diversification dynamics and evolutionary traits across deep time.

Recent advances in computer vision and machine learning, applied to digitized natural history collections, further demonstrate how automated morphological analysis can scale up ecological [69] and macroevolutionary research [75]. For instance, Weeks et al. (2023) developed “Skelevision”, a deep learning workflow capable of automatically segmenting and measuring functional traits from thousands of skeletal specimens [9]. These automated pipelines, supported by a growing ecosystem of open-source tools such as 3D Slicer extensions (e.g., SlicerMorph [18], ALPACA [76]), Biomedisa [8], and custom Python scripts for batch processing (e.g. Buser et al. 2020 [77]), empower researchers to identify macroevolutionary patterns across vast temporal and geographic scales, surpassing the constraints and subjectivity inherent in traditional manual data collection. However, the full potential of these tools depends critically on the availability of large, standardized, and openly accessible training datasets. When 3D morphological data remains scattered across incompatible repositories or locked behind institutional restrictions, these models cannot be trained adequately. This could create a compounding feedback loop: without open data, AI tools for morphometry cannot be developed; without AI tools, large-scale morphological analyses cannot be scaled [14, 78]. This structural dependency between open data and automated morphological analysis represents one of the most pressing challenges facing the field today.

Beyond academic research, the digitization and open sharing of morphological data have also begun to transform institutional areas, including museum collections and education. Projects like oVert [14] illustrate how large-scale imaging initiatives can make rare or fragile specimens accessible to a global community of researchers, educators, and students. High-resolution models produced via CT scanning or photogrammetry can be used in interactive exhibits, digital outreach, or even 3D-printed for classroom use. These resources facilitate learning experiences that are standardized, scalable, and safe for delicate specimens, thereby addressing logistical and pedagogical challenges in teaching disciplines such as palaeontology and evolutionary biology [52].

**Table:** 

Box 2.1 - From remote collections to global resources: The case of French Guiana’s JAGUARS collections	
Digitization is increasingly used to improve access to collections that are often neglected, like those housed in small institutions or located in remote areas with few visitors [79]. In this context, sharing such digital resources online can be a simple and effective way to highlight the natural and cultural heritage of these regions [80, 81]. One example comes from French Guiana, where a recent digitization effort focused on the JAGUARS collections, housed by the wildlife conservation organization Kwata. This collection is representative of the Guiana Plateau region fauna, including skeletons, frozen tissues, and samples preserved in fluid of many Amazonian wild species, offering useful resources for biodiversity research as well as education and knowledge-sharing initiatives. Supported by the Territorial Collectivity of French Guiana, a photogrammetry platform was implemented on site to carry out 3D digitization. This method, known for being quick to implement, portable, and affordable, is particularly suitable for small institutions that often face limited resources [55, 82]. Hundreds of anatomical elements and complete specimens were digitized and made freely available on MorphoSource to date. The 3D models support scientific research and education and serve local wildlife organizations for outreach programs which promote French Guiana’s biodiversity and conservation challenges. Beyond their educational and research value, these digital models also serve as tools for practical conservation work. For example, 3D models can help create identification guidelines used by customs officers, offering a useful resource to help detect illegal wildlife trade [83]. The project, among many others similar initiatives, demonstrates how digital tools can create connections between research, education and conservation activities in regions with high biodiversity and geographical and financial challenges.	

However, this apparent democratization of access through digitization and open sharing must be approached with caution. As Kaiser et al. (2023) emphasize, the institutions that control digitized natural history collections often operate within historical and structural asymmetries rooted in colonialism [84]. Without critical reflection on issues of provenance, representation, and community involvement, digitization risks reinforcing existing power imbalances rather than correcting them. In the context of open access, this raises important ethical questions: who controls the data, who benefits from it, and under what terms? As Hipsley & Sherratt (2019) ask in their section “Whose responsibility is it anyway?”, the answer lies in a shared commitment from individuals, institutions, journals, and funding bodies alike [38]. The rise of AI-based tools adds yet another dimension to this concern: automated pipelines trained predominantly on data from well-resourced institutions will inevitably reflect existing geographic and taxonomic biases, potentially marginalizing the contributions of underrepresented research communities at the computational level [81].These concerns are central to the future of open digital morphology, and highlight the need for inclusive, transparent, and historically aware data-sharing practices [14, 15, 25].

In summary, open-access digital morphology is enabling new modes of inquiry across ecology, palaeontology, and macroevolution, while also expanding the educational and societal reach of natural history collections. Yet its broader impact depends not only on technological innovation, but on the development of equitable frameworks for data sharing, access, and interpretation across both research and institutional domains.

## Key challenges in open access

Despite the rapid adoption of digital imaging in zoology, morphometry, palaeontology, and evolutionary biology, the open sharing of morphological data remains fraught with multifaceted challenges. These range from technical and infrastructural obstacles to ethical and institutional concerns that directly affect the accessibility, quality, and long-term usability of datasets.

A major issue is the lack of standardized protocols for managing and curating 3D morphological data. While platforms like MorphoSource [13] offer structured repositories, persistent challenges include large file sizes, format obsolescence, metadata inconsistencies, and limited interoperability. These technical barriers are further compounded by logistical constraints such as storage limitations and uneven institutional capacity to maintain or preserve high-volume datasets over time.

Ownership and copyright concerns further complicate the open sharing of morphological data. As highlighted by Matsui and Kimura (2022), there is often ambiguity surrounding who holds the rights to 3D reconstructions derived from museum specimens [45]. While fossils themselves are typically not copyrightable, derivative works, such as photogrammetric models or digitally reconstructed skeletons, may qualify as copyrighted content owned by the creator, not the museum. This leads to legal grey areas where models captured in public exhibitions can be uploaded, shared, or even commercialized without institutional oversight. Similarly, Davies et al. (2017) note that disputes between institutions, authors, and funders over data ownership can discourage data deposition and create conflicting obligations regarding open access [25].

Nowadays, many institutions rely on legal tools such as guidelines for proper use, usage licenses, or deposit agreements to retain intellectual property rights over the digital replicas created from their collections [45]. However, there is still legal uncertainty surrounding digital objects created from collection specimens that are not mere digital copies of these items.

This is particularly true for endocasts, which are 3D reconstructions of the internal cavities of structures. In evolutionary biology, the most commonly studied endocasts are probably those of the cranial cavity. In mammals, these cranial endocasts accurately reflect the imprint of the brain and associated structures on the braincase and are therefore highly valuable objects for studying neurological evolution in many extant and fossils lineages [85–87]. Such endocasts are traditionally the result of a long and detailed 3D segmentation process (supported in the last period by automated tools such as those available in 3D Slicer and R) carried out by researchers, leading to the creation of digital objects that differ significantly from the physical specimens preserved in natural history collections [88]. This segmentation process also involves deliberate choices made by the researcher or technician during reconstruction that can be seen as a scientific interpretation, bringing this process closer to creative work. Yet, this kind of creative work is often not addressed by current laws governing the digitization of museum collections in many countries. Nevertheless, it must be emphasized that the creation of such digital objects would simply not be possible without access to the original specimen.

It is noteworthy that 3D segmentation emerged alongside the rise of geometric morphometrics as a pivotal tool in evolutionary biology [39, 89]. Despite this long-standing integration into the field, and notwithstanding several attempts to establish formal guidelines [36, 37, 90], a clear consensus regarding the moral and scientific rights attached to these digital files remains elusive. We believe a balanced approach must recognize both the fundamental role of the physical specimen and the significant intellectual effort required to produce unique 3D models. We therefore advocate for the development of a fair legal framework that accounts for the rights and contributions of both sides.

Within the research community, social and cultural factors also hinder open practices. Competitive pressures, fear of data misuse, and the absence of career recognition for dataset publication contribute to researchers’ reluctance to share raw scans and models [25, 38, 91]. Even when platforms support data sharing, the burden of preparing, documenting, and uploading high-quality datasets, often without institutional reward, falls disproportionately on individual researchers, particularly early-career scholars.

In museum and educational contexts, these issues are exacerbated by the lack of formal data governance policies. As some researchers observed [23, 45], many institutions lack the infrastructure, expertise, or regulatory clarity to manage the digital products they generate or display. This can lead to unauthorized redistribution, misattribution, or even erosion of curatorial authority over digital representations of specimens. It also follows that this uncertainty partially hinders the potential to attract new technological initiatives, made using three-dimensional models [92, 93].

Moreover, as previously discussed in the context of digitization ethics [84], mass digitization efforts often prioritize scale and efficiency over critical engagement with provenance, representation, and inclusivity. Without clear guidelines on who controls data, how it is contextualized, and who benefits from access, digitization risks reinforcing historical and structural asymmetries in knowledge production rather than challenging them.

Finally, while initiatives such as the FAIR data principles (see next section for further details) offer promising frameworks to address many of these challenges, their successful implementation hinges on community-wide consensus and long-term institutional support. In the absence of coherent standards, enforceable rights frameworks, and adequate infrastructure, the full potential of open-access digital morphology remains constrained.

## Potential solutions: FAIR principles, watermarks, and DOIs

Considering the challenges outlined above, including the absence of standards, unclear ownership and copyright frameworks, and the lack of long-term support of digital repositories - several concrete solutions can be proposed to improve the management and sharing of morphological data in zoology, palaeontology, and related fields.

**Fig. 4:**
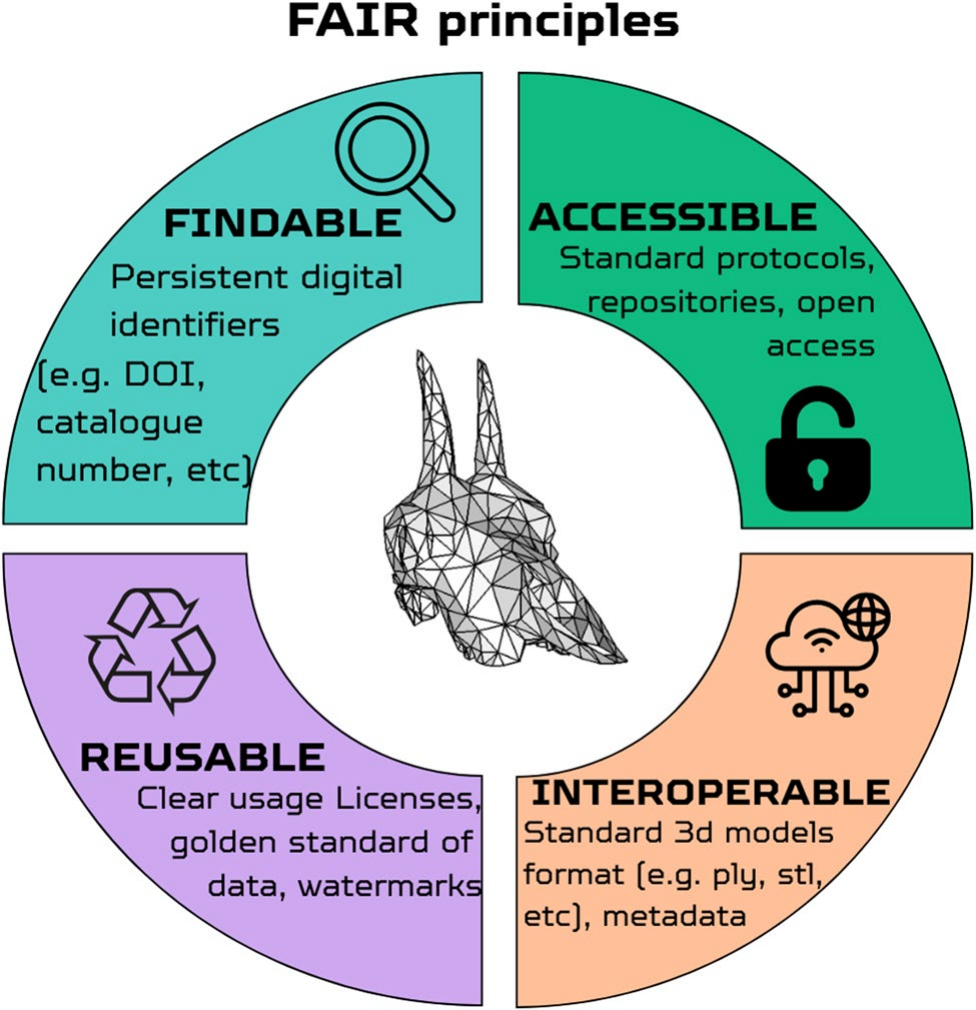
Graphical representation of the FAIR Data Principles, highlighting the essential framework for maximizing the utility and reach of scientific morphological datasets

**Fig. 5:**
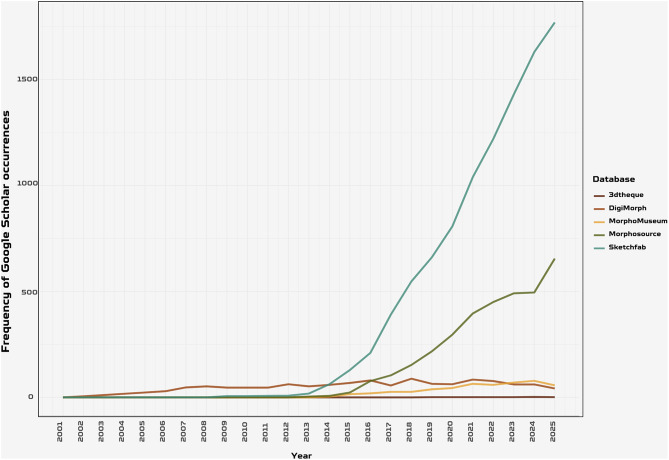
Frequency of Google scholar occurrences for five major 3D morphological data repositories from 2001 to 2025. MorphoSource and Sketchfab emerged as the predominant platforms, with Sketchfab widely adopted by museums for web-based 3D visualization and MorphoSource serving as a specialized repository for biological specimens in academia. Both platforms show substantial growth in recent years

A critical step in this direction is the adoption of the FAIR principles - Findable, Accessible, Interoperable, and Reusable (Fig. 4) [62]. These principles represent a transformative framework for managing and sharing scientific data, with the goal of maximizing its utility for both human and computational stakeholders​. The current data scenario appears to be far from centralization, keeping its fragmented and scarcely integrated nature. While MorphoSource has emerged as the leading dedicated repository for 3D digital morphology data (Fig. 5), the broader data landscape remains fragmented: the number of repositories explicitly hosting 3D data has grown from 26 to 68 between 2015 and 2025 (a 162% increase; [94]) and those mentioning morphology from 19 to 36 (89% increase; [95]), reflecting a proliferation of institutional and discipline-specific platforms that complicates data discovery and reuse. Notably, Sketchfab, despite its high visibility in the literature (Fig. 5), is a commercial platform primarily oriented towards education and public engagement rather than formal scientific archiving and is discussed further below.

This decentralization exacerbates challenges related to data discovery and reusability for both researchers and computational systems (e.g., AI [78]). While several recent publications have advocated for targeted improvements in data management and archival practices [96, 97], the concept of ‘good data management’ remains largely undefined, despite detailed recommendations having been proposed nearly a decade ago [25]. Recently, comprehensive guidelines for the publication of 3D datasets in palaeontology have been proposed [98] emphasizing the importance of standardized metadata. Such contributions highlight a growing consensus within the scientific community on the urgent need for uniform standards to ensure better management and long-term utility of digital data.

Developed to enhance the reusability of scholarly data, FAIR principles serve as a foundational guide for data producers and publishers. In biology and zoology, the adoption of such principles could address several persistent challenges in open data, including inconsistencies in data standards, limited accessibility, and difficulties in cross-disciplinary integration.

Findability ensures that datasets are indexed using globally unique and persistent identifiers, supported by metadata that allow effective searchability across repositories and disciplines. This remains particularly challenging in 3D morphology, where no universal metadata standard exists across imaging platforms [37]. Accessibility guarantees that data can be retrieved using standardized communication protocols, even when access requires authentication. Interoperability calls for shared vocabularies, metadata standards, and data formats that enable seamless integration across systems (a goal currently hampered by the proliferation of semi-proprietary rendering formats such as AM, TXM, and VGL, which cannot be opened across multiple software platforms [37]). Only highly derived final products such as STL or PLY meshes are standardized for cross-platform use, meaning that the most scientifically valuable intermediate data remains effectively locked within specific software ecosystems. Reusability, perhaps the most critical feature in a context where datasets are often repurposed for different questions, emphasizes transparent usage licenses and clear documentation of provenance and data quality [62]. This dimension has acquired new urgency with the rise of AI-based tools for automated segmentation and landmarking, which require large, standardized, and openly licensed training datasets, making restrictive or ambiguous licensing not merely an inconvenience, but a direct impediment to the field’s computational future.

For example, repositories adhering to FAIR principles, such as the FAIRsharing [62] registry or the already cited GenBank, play a crucial role in this landscape by systematically cataloguing data standards, including versioning where applicable, thereby facilitating the adoption of uniform practices across scientific communities. These critical infrastructures are continuously curated and refined to enhance scholarly output, support both human and machine users, and provide sophisticated tools for accessing content in rich, dynamic ways.

Applying these principles to digital morphological datasets in zoology and palaeontology could yield similar benefits, promoting data integration across disciplines and enhancing reproducibility. Moreover, the emphasis on machine-actionable data aligns with modern research needs, facilitating automated analyses and enabling large-scale meta-studies​.

By adopting FAIR principles in zoology, palaeontology, anthropology, and biology in general, researchers can transform the current fragmented data landscape into a more cohesive and productive ecosystem. This shift would not only enhance data transparency and accessibility but also accelerate innovation, fostering a more collaborative and efficient scientific community.

While platforms like Sketchfab are excellent for educational purposes and public engagement (e.g., the D’Arcy Thompson Zoology Museum collection), they are less suited for long-term research data storage due to their commercial nature and lack of guaranteed data longevity. In parallel, the use of public, open-access repositories, more consolidated inside academic world, such as MorphoSource [13], Dryad, and Figshare presents a pragmatic way to adopt FAIR-aligned practices while minimizing technical and financial burdens. These platforms often provide contributors with interfaces for managing metadata, applying licensing restrictions, and tracking usage. Importantly, they offer scalable infrastructure that addresses the issue of repository sustainability, enabling institutions to share high-resolution 3D data without maintaining their own servers. By encouraging deposit in such platforms, the community can begin to build interoperable, cross-referenced archives that remain accessible and traceable over time.

To further address the problem of data attribution and citation, another essential step is the creation of persistent digital identifiers, modelled on Digital Object Identifier (DOIs) [99], that can be assigned to individual digital specimens. These identifiers could integrate existing museum catalogue numbers and be incorporated into repository metadata (e.g., Zenodo and Figshare give free repositories DOI), thus enabling the direct and standardized citation of specimens across studies and publications. This system would not only support reproducibility but also ensure that museums and digitization teams receive proper credit for their contributions.

Complementing this system, recent advances in invisible digital watermarking offer an additional layer of traceability. These techniques allow for the embedding of institutional identifiers, catalogue numbers, and authorship metadata directly within the geometric or texture data of 3D models [100, 101]. Applied to digital morphology, such watermarks could act as embedded citations, preserving the link between dataset and institution even when models are detached from their source repositories or redistributed. For museum-generated models in particular, watermarking may help mitigate risks of unauthorized reuse or commercial exploitation, while also facilitating appropriate citation in scholarly publications.

In summary, the integration of FAIR principles, adoption of scalable repositories, use of persistent identifiers, and implementation of watermarking represent complementary solutions to the key obstacles facing open digital morphology. Each addresses a specific layer of the problem: from data structure and repository management, to authorship, legal attribution, and ethical reuse. To fully realize the promise of open access in morphology, technical and policy solutions must be implemented in tandem with a cultural shift toward responsible data stewardship and collaborative infrastructure development.

## Conclusions

The integration of digital morphological techniques and open-access infrastructures has introduced a paradigm shift in zoological, paleontological, and evolutionary research. These successes highlight the available potential for similar practices in digital morphology, where systematic data sharing could yield comparable benefits for biodiversity research, evolutionary studies, and applied sciences. High-resolution 3D datasets offer unprecedented opportunities for comparative analyses, functional modelling, and educational outreach, enabling new forms of interdisciplinary collaboration and reproducible science [5, 12, 23, 35, 36]. However, this technological potential is accompanied by persistent challenges. Issues of standardization, long-term repository sustainability, unclear copyright frameworks, and inconsistent attribution practices continue to limit the effective dissemination and reuse of morphological data [12, 16, 18, 22, 40, 42, 91].

As this review has outlined, implementing practical solutions such as the FAIR principles [36, 43, 62], persistent digital identifiers [102], and invisible watermarking [52, 53] could significantly enhance data findability, traceability, and reusability. Public and open-access repositories provide valuable infrastructure for sharing models in accessible formats; while watermarking and unique IDs help ensure proper recognition of contributors and institutions. Yet, these technical interventions must be matched by institutional and cultural shifts toward transparent policies, ethical data governance, and academic systems that value open data contributions alongside traditional publications.

Ultimately, the future of open-access digital morphology will depend on the community’s ability to balance openness with responsibility, scale with sustainability, and innovation with equity. By embracing this balance, researchers and institutions can foster a more integrated, ethical, and inclusive scientific ecosystem—one in which morphological knowledge is not only generated but also preserved, shared, and enriched across generations.

## Data Availability

Data sharing is not applicable to this article as no datasets were generated or analysed during the current study. All data discussed or cited are available within the article and its included tables and figures.

## References

[1] Lautenschlager S. Multibody dynamics analysis as a computational tool to reconstruct the function and palaeobiology of extinct organisms. Palaeontology. 2020;63(5):703–15. 10.1111/pala.12501.

[2] Douglas-Jones R, Walford A, Seaver N, Introduction. Towards an anthropology of data. J Roy Anthropol Inst. 2021;27:9–25. 10.1111/1467-9655.13477.

[3] Sutton M, Rahman I, Garwood R. Techniques for virtual palaeontology. Wiley; 2014.

[4] Ford KL, Albert JS, Summers AP, Hedrick BP, Schachner ER, Jones AS, Evans K, Chakrabarty P. A New Era of Morphological Investigations: Reviewing Methods for Comparative Anatomical Studies. Integr Org Biol. 2023;5(1):obad008. 10.1093/iob/obad008. PMID: 37035037; PMCID: PMC10081917. 37035037 10.1093/iob/obad008 PMC10081917

[5] Goswami A, Clavel J. Morphological evolution in a time of phenomics. Paleobiology. 2025;51:195–213. 10.1017/pab.2024.35.

[6] Carlson WD, Rowe T, Ketcham RA, Colbert MW. Applications of high-resolution X-ray computed tomography in petrology, meteoritics and palaeontology. SP. 2003;215:7–22. 10.1144/GSL.SP.2003.215.01.02.

[7] Subburaj K, Ravi B, Agarwal M. Automated identification of anatomical landmarks on 3D bone models reconstructed from CT scan images. Comput Med Imaging Graph. 2009;33:359–68. 10.1016/j.compmedimag.2009.03.001. 19345065 10.1016/j.compmedimag.2009.03.001

[8] Lösel PD, Van De Kamp T, Jayme A, Ershov A, Faragó T, Pichler O, et al. Introducing Biomedisa as an open-source online platform for biomedical image segmentation. Nat Commun. 2020;11:5577. 10.1038/s41467-020-19303-w. 33149150 10.1038/s41467-020-19303-w PMC7642381

[9] Weeks BC, Zhou Z, O’Brien BK, Darling R, Dean M, Dias T, et al. A deep neural network for high-throughput measurement of functional traits on museum skeletal specimens. Methods Ecol Evol. 2023;14:347–59. 10.1111/2041-210X.13864.

[10] Didziokas M, Pauws E, Kölby L, Khonsari RH, Moazen M. BounTI (boundary-preserving threshold iteration): A user‐friendly tool for automatic hard tissue segmentation. J Anat. 2024;245:829–41. 10.1111/joa.14063. 38760955 10.1111/joa.14063 PMC11547236

[11] He Y, Camaiti M, Roberts LE, Mulqueeney JM, Ioannou E, Didziokas M et al. SPROUT: A User-friendly, Scalable Toolkit for Multi-class Segmentation of Volumetric Images. 2024. 10.1101/2024.11.22.624847

[12] Lebrun R, Orliac MJ, MORPHOMUSEUM: AN ONLINE PLATFORM FOR PUBLICATION AND STORAGE OF VIRTUAL SPECIMENS. Paleontol Soc Pap. 2016;22:183–95. 10.1017/scs.2017.14.

[13] Boyer DM, Gunnell GF, Kaufman S, McGeary TM. Morphosource: archiving and sharing 3D digital specimen. Paleontol Soc Pap. 2016;22:157–81. 10.1017/scs.2017.13.

[14] Blackburn DC, Boyer DM, Gray JA, Winchester J, Bates JM, Baumgart SL, et al. Increasing the impact of vertebrate scientific collections through 3D imaging: The openVertebrate (oVert) Thematic Collections Network. Bioscience. 2024;74:169–86. 10.1093/biosci/biad120. 38560620 10.1093/biosci/biad120 PMC10977868

[15] Cicero C, Koo MS, Braker E, Abbott J, Bloom D, Campbell M, et al. Arctos: Community-driven innovations for managing natural and cultural history collections. PLoS ONE. 2024;19:e0296478. 10.1371/journal.pone.0296478. 38820381 10.1371/journal.pone.0296478 PMC11142579

[16] Clavel J, Escarguel G, Merceron G. mv morph: an r package for fitting multivariate evolutionary models to morphometric data. Methods Ecol Evol. 2015;6:1311–9. 10.1111/2041-210X.12420.

[17] Baken EK, Collyer ML, Kaliontzopoulou A, Adams DC. geomorph v4.0 and gmShiny: enhanced analytics and a new graphical interface for a comprehensive morphometric experience. Methods Ecol Evol. 2021;12:2355–63.

[18] Rolfe S, Pieper S, Porto A, Diamond K, Winchester J, Shan S, et al. SlicerMorph: An open and extensible platform to retrieve, visualize and analyse 3D morphology. Methods Ecol Evol. 2021;12:1816–25. 10.1111/2041-210X.13669. 40401087 10.1111/2041-210x.13669 PMC12094517

[19] Rostamian R, Shariat Panahi M, Karimpour M, Kashani HG, Abi A. A deep learning-based multi-view approach to automatic 3D landmarking and deformity assessment of lower limb. Sci Rep. 2025;15:534. 10.1038/s41598-024-84387-z. 39747979 10.1038/s41598-024-84387-z PMC11697423

[20] Anné J. Interpreting pathologies in extant and extinct archosaurs using micro-CT. PeerJ. 2015;3. 10.7717/peerj.1130. 10.7717/peerj.1130 PMC4525691 26246971

[21] Qing X, Bert W. 3D printing in zoological systematics: Integrative taxonomy of Labrys chinensis gen. nov., sp. nov. (Nematoda: Tylenchomorpha). J Zool Syst Evol Res. 2018;56:35–47. 10.1111/jzs.12191.

[22] Cardini A, Chiapelli M. How flat can a horse be? Exploring 2D approximations of 3D crania in equids. Zoology. 2020;139:125746. 10.1016/j.zool.2020.125746. 32086141 10.1016/j.zool.2020.125746

[23] Ziegler MJ, Perez VJ, Pirlo J, Narducci RE, Moran SM, Selba MC, et al. Applications of 3D Paleontological Data at the Florida Museum of Natural History. Front Earth Sci. 2020;8:600696. 10.3389/feart.2020.600696.

[24] Ford KL, Albert JS, Summers AP, Hedrick BP, Schachner ER, Jones AS, et al. A New Era of Morphological Investigations: Reviewing Methods for Comparative Anatomical Studies. Integr Organismal Biology. 2023;5:obad008. 10.1093/iob/obad008. 10.1093/iob/obad008 PMC10081917 37035037

[25] Davies TG, Rahman IA, Lautenschlager S, Cunningham JA, Asher RJ, Barrett PM, et al. Open data and digital morphology. Proc R Soc B. 2017;284:20170194. 10.1098/rspb.2017.0194. 28404779 10.1098/rspb.2017.0194 PMC5394671

[26] Polly PD. Geometric Morphometrics. In: López Varela SL, editor. The Encyclopedia of Archaeological Sciences. 1st edition. Wiley; 2018. pp. 1–5. 10.1002/9781119188230.saseas0258

[27] Maga A. Digital Morphology: The Final Frontier. iar. 2023;0:0–0. 10.26650/IAR2022-1174374.

[28] Anas I, Bamgbose B, Nuhu S. A comparison between 2D and 3D methods of quantifying facial morphology. Heliyon. 2019;5:e01880. 10.1016/j.heliyon.2019.e01880. 31338446 10.1016/j.heliyon.2019.e01880 PMC6579906

[29] Chatar N, Fischer V, Siliceo G, Antón M, Morales J, Salesa MJ. Morphometric Analysis of the Mandible of Primitive Sabertoothed Felids from the late Miocene of Spain. J Mammal Evol. 2021;28:753–71. 10.1007/s10914-021-09541-0.

[30] De Leo N, Chimenti C, Maiorano L, Tamagnini D. Protocol for 3D photogrammetry and morphological digitization of complex skulls. STAR Protocols. 2025;6:103572. 10.1016/j.xpro.2024.103572. 39826113 10.1016/j.xpro.2024.103572 PMC11787561

[31] Cardini A. Missing the third dimension in geometric morphometrics: how to assess if 2D images really are a good proxy for 3D structures? Hystrix Italian J Mammalogy. 2014;25(2):73–81. 10.4404/hystrix-25.2-10993.

[32] Andrea C, Marika C. How flat can a horse be? Exploring 2D approximations of 3D crania in equids. Zoology. Volume 139. 2020. 125746. ISSN 0944–2006. 10.1016/j.zool.2020.125746 10.1016/j.zool.2020.125746 32086141

[33] Zhu Y. Open-access policy and data-sharing practice in UK academia. J Inform Sci. 2020;46:41–52. 10.1177/0165551518823174.

[34] Grzenda M, Legierski J. Inf Syst Front. 2021;23:495–513. 10.1007/s10796-019-09954-6. Towards Increased Understanding of Open Data Use for Software Development.

[35] Johnson KG, Filkorn HF, Stecheson M. A collaborative system for sharing paleontology collections data. Geosphere. 2005;1:61. 10.1130/GES00011.1.

[36] Mulligan CJ, Boyer DM, Turner TR, Delson E, Leonard WR. Data sharing in biological anthropology. Am J Biol Anthropol. 2022;178:26–53. 10.1002/ajpa.24499.

[37] Gignac PM, Aceves V, Baker S, Barnes JJ, Bell J, Boyer D, et al. The role of networks to overcome large-scale challenges in tomography: The non-clinical tomography users research network. Tomography Mater Struct. 2024;5:100031. 10.1016/j.tmater.2024.100031.

[38] Hipsley CA, Sherratt E. Psychology, not technology, is our biggest challenge to open digital morphology data. Sci Data. 2019;6. 10.1038/s41597-019-0047-0. 41. 10.1038/s41597-019-0047-0 PMC6486585 31028285

[39] Mitteroecker P, Schaefer K. Thirty years of geometric morphometrics: Achievements, challenges, and the ongoing quest for biological meaningfulness. Am J Biol Anthropol. 2022;178:181–210. 10.1002/ajpa.24531. 36790612 10.1002/ajpa.24531 PMC9545184

[40] Benson DA, Clark K, Karsch-Mizrachi I, Lipman DJ, Ostell J, Sayers EW, GenBank. Nucl Acids Res. 2014;42:D32–7. 10.1093/nar/gkt1030. 24217914 10.1093/nar/gkt1030 PMC3965104

[41] Hood L, Rowen L. The human genome project: big science transforms biology and medicine. Genome Med. 2013;5:79. 10.1186/gm483. 24040834 10.1186/gm483 PMC4066586

[42] Mangul S, Martin LS, Langmead B, Sanchez-Galan JE, Toma I, Hormozdiari F, et al. How bioinformatics and open data can boost basic science in countries and universities with limited resources. Nat Biotechnol. 2019;37:324–6. 10.1038/s41587-019-0053-y. 30833765 10.1038/s41587-019-0053-y

[43] Conroy M, Sellors J, Effingham M, Littlejohns TJ, Boultwood C, Gillions L, et al. The advantages of UK Biobank’s open-access strategy for health research. J Intern Med. 2019;286:389–97. 10.1111/joim.12955. 31283063 10.1111/joim.12955 PMC6790705

[44] Dowding EM, Dunne EM, Collins KS, et al. The billion-dollar case for sustaining palaeontology’s digital databases. Nat Ecol Evol. 2026;10:594–605. 10.1038/s41559-026-02985-8. 41667741 10.1038/s41559-026-02985-8 PMC12971485

[45] Matsui K, Kimura Y. Museum Exhibitions of Fossil Specimens Into Commercial Products: Unexpected Outflow of 3D Models due to Unwritten Image Policies. Front Earth Sci. 2022;10:874736. 10.3389/feart.2022.874736.

[46] Buser TJ, Sidlauskas BL, Summers AP. 2D or Not 2D? Testing the Utility of 2D Vs. 3D Landmark Data in Geometric Morphometrics of the Sculpin Subfamily Oligocottinae (Pisces; Cottoidea). Anat Rec. 2018;301:806–18. 10.1002/ar.23752. 10.1002/ar.23752 29244247

[47] Hublin J-J. Free digital scans of human fossils. Nature. 2013;497:183–183. 10.1038/497183a. 23657334 10.1038/497183a

[48] Lombardi M. Sustainability of 3D heritage data: life cycle and impact. AeC. 2023;34:339–56. 10.19282/ac.34.2.2023.18.

[49] Peukert C, Windisch M. The economics of copyright in the digital age. J Economic Surveys. 2025;39:877–903. 10.1111/joes.12632.

[50] da Silva AG, de Oliveira RP, de Oliveira Bastos C, de Carvalho EA, Gomes BD. A mobile hybrid deep learning approach for classifying 3D-like representations of Amazonian lizards. Front Artif Intell 8:15243802025. 10.3389/frai.2025.1524380 10.3389/frai.2025.1524380 PMC12378728 40873492

[51] Loy A, Slice DE. Image data banks and geometric morphometrics. In P. L. Nimis & R. Vignes Lebbe, editors, Tools for identifying biodiversity: Progress and problems. EUT Edizioni Università di Trieste. 2010. 243–248.

[52] Cunningham JA, Rahman IA, Lautenschlager S, Rayfield EJ, Donoghue PCJ. A virtual world of paleontology. Trends Ecol Evol. 2014;29:347–57. 10.1016/j.tree.2014.04.004. 24821516 10.1016/j.tree.2014.04.004

[53] Otero A, Pérez Moreno A, Falkingham P, Cassini G, Ruella A, Militello M et al. THREE-DIMENSIONAL IMAGE SURFACE ACQUISITION IN VERTEBRATE PALEONTOLOGY: A REVIEW OF PRINCIPAL TECHNIQUES. PEAPA. 2020. 10.5710/PEAPA.04.04.2020.310

[54] Sutton MD, Rahman IA, Garwood RJ, editors. Techniques for Virtual Palaeontology. 1st edition. Wiley; 2013. 10.1002/9781118591192

[55] Falkingham P. Acquisition of high resolution three-dimensional models using free, open-source, photogrammetric software. Palaeontologia Electronica. 2012. 10.26879/264.

[56] Mallison H, Wings O. Photogrammetry in Paleontology - a Practical Guide. J Paleontological Techniques. 2014;12:1–31.

[57] Konstantakis M, Trichopoulos G, Aliprantis J, Michalakis K, Caridakis G, Thanou A, et al. An Enhanced Methodology for Creating Digital Twins within a Paleontological Museum Using Photogrammetry and Laser Scanning Techniques. Heritage. 2023;6:5967–80. 10.3390/heritage6090314.

[58] Cottrell B, Kalacska M, Arroyo-Mora JP, Lucanus O, Cottrell P, Lehnhart T, et al. 3D reconstructions of stranded marine mammals via easily accessible remote sensing tools for use in morphometrics and visualizations. Front Mar Sci. 2025;12:1485788. 10.3389/fmars.2025.1485788.

[59] Polly P, MacLeod N. Locomotion in fossil Carnivora: An application of eigensurface analysis for morphometric comparison of 3D surfaces. Palaeontol Electron. 2008;11.

[60] Slizewski A, Astrid, Semal P, Patrick. Experiences with low and high cost 3D surface scanner. Quartär. 2009;56:131–8.

[61] Abel R, Laurini C, Richter M. A palaeobiologist’s guide to virtual micro-CT preparation. Palaeontologia Electronica. 2012. 10.26879/284.

[62] Wilkinson MD, Dumontier M, Aalbersberg IJ, Appleton G, Axton M, Baak A, et al. The FAIR Guiding Principles for scientific data management and stewardship. Sci Data. 2016;3:160018. 10.1038/sdata.2016.18. 26978244 10.1038/sdata.2016.18 PMC4792175

[63] Modesto-Mata M, Thiebaut A, Krueger KL, Maga AM, Joganic JL, Ryan TM, et al. Easier said than done: unexpected hurdles to preparing ∼1,000 cranial CT scans for data collection from an online digital repository. PeerJ. 2025;13:e20172. 10.7717/peerj.20172. 41142304 10.7717/peerj.20172 PMC12548635

[64] Bernardini F, Rushmeier H. The 3D Model Acquisition Pipeline. Comput Graphics Forum. 2002;21:149–72. 10.1111/1467-8659.00574.

[65] Tocheri MW. Laser Scanning: 3D Analysis of Biological Surfaces. In: Sensen CW, Hallgrímsson B, editors. Advanced Imaging in Biology and Medicine. Berlin, Heidelberg: Springer Berlin Heidelberg; 2009. pp. 85–101. 10.1007/978-3-540-68993-5_4.

[66] Geng J. Structured-light 3D surface imaging: a tutorial. Adv Opt Photon. 2011;3:128. 10.1364/AOP.3.000128.

[67] Fischer V, Vaczi N, Bennion RF, Cottereau R, Lawrence Wujek J, MacLaren JA. Accurate specimen digitization through glass achieved and validated using 3D surface scanning. Palaeontol Electron. 2024. 10.26879/1375.

[68] Walker M, Humphries S. 3D Printing: Applications in evolution and ecology. Ecol Evol. 2019;9:4289–301. 10.1002/ece3.5050. 31016005 10.1002/ece3.5050 PMC6468079

[69] Wilson RJ, De Siqueira AF, Brooks SJ, Price BW, Simon LM, Van Der Walt SJ, et al. Applying computer vision to digitised natural history collections for climate change research: Temperature-size responses in British butterflies. Methods Ecol Evol. 2023;14:372–84. 10.1111/2041-210X.13844.

[70] Watson CM, Francis GR. Three dimensional printing as an effective method of producing anatomically accurate models for studies in thermal ecology. J Therm Biol. 2015;51:42–6. 10.1016/j.jtherbio.2015.03.004. 25965016 10.1016/j.jtherbio.2015.03.004

[71] Leiva FP, Ellers J, Berg MP, Cuxart-Erruz R, Barneche DR, Blackburn TM, et al. ShareTrait: Towards interoperable and reusable individual trait‐based data in ectotherms. Funct Ecol. 2025;39:3124–38. 10.1111/1365-2435.70147.

[72] MacLean HJ, Nielsen ME, Kingsolver JG, Buckley LB. Using museum specimens to track morphological shifts through climate change. Phil Trans R Soc B. 2018;374:20170404. 10.1098/rstb.2017.0404. 30455218 10.1098/rstb.2017.0404 PMC6282086

[73] Cornwell W, Nakagawa S. Phylogenetic comparative methods. Curr Biol. 2017;27:R333–6. 10.1016/j.cub.2017.03.049. 28486113 10.1016/j.cub.2017.03.049

[74] Jeiter J, Smets E. Integrating comparative morphology and development into evolutionary research. Taxon. 2023;72:724–32. 10.1002/tax.12983.

[75] He Y, Mulqueeney JM, Watt EC, Salili-James A, Barber NS, Camaiti M, et al. Opportunities and Challenges in Applying AI to Evolutionary Morphology. Integr Organismal Biology. 2024;6:obae036. 10.1093/iob/obae036. 10.1093/iob/obae036 PMC12082097 40433986

[76] Porto A, Rolfe S, Maga AM. ALPACA: A fast and accurate computer vision approach for automated landmarking of three-dimensional biological structures. Methods Ecol Evol. 2021;12(11):2129–44. 10.1111/2041-210X.13689. Epub 2021 Aug 9. PMID: 35874971; PMCID: PMC9291522. 35874971 10.1111/2041-210X.13689 PMC9291522

[77] Buser TJ, Boyd OF, Cortés Á, Donatelli CM, Kolmann MA et al. The Natural Historian’s Guide to the CT Galaxy: Step-by-Step Instructions for Preparing and Analyzing Computed Tomographic (CT) Data Using Cross-Platform, Open Access Software, Integrative Organismal Biology, Volume 2, Issue 1, 2020. 10.1093/iob/obaa009 10.1093/iob/obaa009 PMC7671151 33791553

[78] Hassoun S, Jefferson F, Shi X, Stucky B, Wang J, Rosa E. Artificial Intelligence for Biology. Integr Comp Biol. 2022;61:2267–75. 10.1093/icb/icab188. 34448841 10.1093/icb/icab188

[79] Beaman R, Cellinese N. Mass digitization of scientific collections: New opportunities to transform the use of biological specimens and underwrite biodiversity science. ZK. 2012;209:7–17. 10.3897/zookeys.209.3313. 10.3897/zookeys.209.3313 PMC3406463 22859875

[80] Nelson G, Ellis S. The history and impact of digitization and digital data mobilization on biodiversity research. Phil Trans R Soc B. 2019;374:20170391. 10.1098/rstb.2017.0391. 10.1098/rstb.2017.0391 PMC6282090 30455209

[81] Drew JA, Moreau CS, Stiassny MLJ. Digitization of museum collections holds the potential to enhance researcher diversity. Nat Ecol Evol. 2017;1:1789–90. 10.1038/s41559-017-0401-6. 29133899 10.1038/s41559-017-0401-6

[82] Medina JJ, Maley JM, Sannapareddy S, Medina NN, Gilman CM, McCormack JE. A rapid and cost-effective pipeline for digitization of museum specimens with 3D photogrammetry. PLoS ONE. 2020;15:e0236417. 10.1371/journal.pone.0236417. 32790700 10.1371/journal.pone.0236417 PMC7425849

[83] Pirotta V, Shen K, Liu S, Phan HTH, O’Brien JK, Meagher P, et al. Detecting illegal wildlife trafficking via real time tomography 3D X-ray imaging and automated algorithms. Front Conserv Sci. 2022;3:757950. 10.3389/fcosc.2022.757950.

[84] Kaiser K, Heumann I, Nadim T, Keysar H, Petersen M, Korun M, et al. Promises of mass digitisation and the colonial realities of natural history collections. s J Nat Sci Collections. 2023;11:13–25.

[85] Balanoff AM, Bever GS. The Role of Endocasts in the Study of Brain Evolution. Evolution of Nervous Systems. Elsevier; 2017. pp. 223–41. 10.1016/B978-0-12-804042-3.00023-3.

[86] De Sousa AA, Beaudet A, Calvey T, Bardo A, Benoit J, Charvet CJ, et al. From fossils to mind. Commun Biol. 2023;6:636. 10.1038/s42003-023-04803-4. 37311857 10.1038/s42003-023-04803-4 PMC10262152

[87] Bertrand OC, Michaud M, Kirk EC. How the neurosensory system provides clues for the adaptive radiation of mammals. Reference Module in Neuroscience and Biobehavioral Psychology. Elsevier; 2025. p. B9780443273803000191. 10.1016/B978-0-443-27380-3.00019-1.

[88] Balanoff AM, Bever GS, Colbert MW, Clarke JA, Field DJ, Gignac PM, et al. Best practices for digitally constructing endocranial casts: examples from birds and their dinosaurian relatives. J Anat. 2016;229:173–90. 10.1111/joa.12378. 26403623 10.1111/joa.12378 PMC4948053

[89] Conroy GC, Vannier MW. Noninvasive Three-Dimensional Computer Imaging of Matrix-Filled Fossil Skulls by High-Resolution Computed Tomography. Science. 1984;226:456–8. 10.1126/science.226.4673.456. 17799939 10.1126/science.226.4673.456

[90] Storeide MSB, George S, Sole A, Hardeberg JY. Standardization of digitized heritage: a review of implementations of 3D in cultural heritage. Herit Sci. 2023;11:249. 10.1186/s40494-023-01079-z.

[91] Gomes DGE, Pottier P, Crystal-Ornelas R, Hudgins EJ, Foroughirad V, Sánchez-Reyes LL, et al. Why don’t we share data and code? Perceived barriers and benefits to public archiving practices. Proc R Soc B. 2022;289:20221113. 10.1098/rspb.2022.1113. 36416041 10.1098/rspb.2022.1113 PMC9682438

[92] Herraiz JL, Villena JA, Vilaplana-Climent A, Conejero N, Cocera H, Botella H, et al. The palaeontological virtual collection of the University of Valencia’s Natural History Museum: a new tool for palaeontological heritage outreach. Span J Palaeontol. 2019;34:139–44. 10.7203/sjp.34.1.15249.

[93] Delgado J, West R, Barmpoutis A, Jang SH, Stanley E, Kang H. Enhancing Museum Experience with VR by Situating 3D Collections in Contex. In: Proceedings of the 23rd Annual ACM Interaction Design and Children Conference. Delft Netherlands: ACM; 2024. pp. 670–5. 10.1145/3628516.3659372

[94] https://www.re3data.org/metrics/repositoriesNumber/search?query=3d

[95] https://www.re3data.org/metrics/repositoriesNumber/search?query=morphology

[96] White E, Baldridge E, Brym Z, Locey K, McGlinn D, Supp S. Nine simple ways to make it easier to (re)use your data. IEE. 2013. 10.4033/iee.2013.6b.6.f. 6.

[97] Roche DG, Kruuk LEB, Lanfear R, Binning SA. Public Data Archiving in Ecology and Evolution: How Well Are We Doing? PLoS Biol. 2015;13:e1002295. 10.1371/journal.pbio.1002295. 26556502 10.1371/journal.pbio.1002295 PMC4640582

[98] Díez Díaz V, Belvedere M, Depraetere M, Holwerda F, Schwarz D. How should I publish my digital fossil? Recommendations for the publication of comprehensive 3D datasets in palaeontological studies. Palaeontology. 2026;69:e70052. 10.1111/pala.70052.

[99] Basson I, Simard M-A, Ouangré ZA, Sugimoto CR, Larivière V. The effect of data sources on the measurement of open access: A comparison of Dimensions and the Web of Science. PLoS ONE. 2022;17:e0265545. 10.1371/journal.pone.0265545. 35358227 10.1371/journal.pone.0265545 PMC8970383

[100] Narendra M, Valarmathi ML, Anbarasi LJ. Watermarking techniques for three-dimensional (3D) mesh models: a survey. Multimedia Syst. 2022;28:623–41. 10.1007/s00530-021-00860-z.

[101] Shaliyar M, Mustafa K. Watermarking approach for source authentication of web content in online social media: a systematic literature review. Multimed Tools Appl. 2023;83:54027–79. 10.1007/s11042-023-17559-0.

[102] Mondal H, Mondal S. Digital object identifier: What it is and why it matters? IJSA. 2023;2:77–80. 10.25259/IJSA_20_2023.

